# Antineoplastic effects of auranofin in human pancreatic adenocarcinoma preclinical models

**DOI:** 10.1016/j.sopen.2019.05.004

**Published:** 2019-07-03

**Authors:** Mayrim V. Rios Perez, David Roife, Bingbing Dai, Michael Pratt, Ryszard Dobrowolski, Ya'an Kang, Xinqun Li, Jithesh J. Augustine, Rafal Zielinski, Waldemar Priebe, Jason B. Fleming

**Affiliations:** a Department of General Surgery, University of Puerto Rico, Medical Sciences Campus, San Juan, Puerto Rico 00936; b Department of Gastrointestinal Oncology, H Lee Moffitt Cancer Center and Research Institute, Tampa, FL 33612; c Department of Surgical Oncology, The University of Texas MD Anderson Cancer Center, 1515 Holcombe Blvd., Unit 107, Houston, TX 77030; d School of Medicine, Baylor College of Medicine, Houston, TX 77030; e Department of Analytical Chemistry and Instrumental Analysis, Maria Curie-Sklodowska University Sq. 3, 20-031, Lublin, Poland; f Department of Experimental Therapeutics, The University of Texas, MD Anderson Cancer Center, 1901 East Road, Unit 1950, Houston, TX 77054

## Abstract

**Background:**

Auranofin, a Food and Drug Administration–approved anti-rheumatic agent with anticancer properties for lung and ovarian cancer, has never been studied for pancreatic cancer. We hypothesize that auranofin may prevent pancreatic ductal adenocarcinoma progression by inhibition of Txnrd1 and HIF-1α.

**Methods:**

*In vitro* sensitivity of human pancreatic ductal adenocarcinoma cell lines was determined based on IC50. Western blot assays were used to interrogate mechanisms of apoptosis and resistance. *Ex vivo* live tissue slice assays of xenografts allowed for testing of a larger number of PDX samples with high efficiency. *In vivo* pancreatic ductal adenocarcinoma orthotopic mouse models using MiaPaCa-2 Luc + cells were designed to determine optimal dose and antitumor effect.

**Results:**

We found that 10 of 15 tested pancreatic ductal adenocarcinoma cell lines were sensitive to auranofin based on IC50s below 5 μmol/L. *Ex vivo* tissue growth inhibition greater than 44% was observed for 13 PDX tissue cases treated with 10 μmol/L auranofin. High Txnrd1 expression was observed for resistant cell lines. *In vivo* studies showed 15 mg/kg IP as the optimal dose with absence of gross solid organ metastasis up to 13 weeks post-treatment (median survival 8 and 12 weeks, respectively; *P* = .0953).

**Conclusions:**

We have demonstrated that auranofin prevents pancreatic ductal adenocarcinoma progression using multiple models. Our study suggests inhibition of Txnrd1 and HIF-1α as possible mechanisms of action, and Txnrd1 as a biomarker of resistance. Based on these data, an off-label Phase 0 clinical trial with this FDA-approved drug should be considered for patients with pancreatic cancer.

## **INTRODUCTION**

Pancreatic ductal adenocarcinoma (PDAC) remains one of the most deadly types of cancer with a worldwide incidence of 1–10 for every 100,000, and steadily low survival rates over the last 3 decades, in part due to failure of tumor response to available treatment regimens [1], [2], [3]. Auranofin, an organic gold compound FDA-approved for rheumatoid arthritis, has attracted interest due to anticancer properties discovered in studies in leukemia, lymphoma, bone, lung, ovarian, gastric, colorectal, melanoma and breast malignancies [4], [5], [6], [7], [8], [9], [10], [11], [12], [13]. Throughout the last 3 decades of research, multiple mechanisms of action have been ascribed to auranofin for its anti-cancer effects, including: inhibition of Txnrd1 (thioredoxin reductase 1, aka Trx1 and Trxr1), a major intracellular glutathione-like reducing system, cytotoxic increase in reactive oxygen species, disruption of mitochondrial membrane potential, trigger of endoplasmic reticulum stress and activation of caspase, and inhibition of ubiquitin-proteasome system [5], [11], [14], [15], [16], [17]. Inhibitors of the thioredoxin redox system have been implicated in the inhibition of pro-angiogenic effectors, hypoxia-inducible factor-1 α (HIF1α) and vascular endothelial growth factor (VEGF), both of which are overexpressed in many malignancies including pancreatic cancer [18], [19], [20]. Despite the data available in other cancer models, the role of auranofin as a therapeutic against pancreatic cancer has been understudied, with one report many years prior [21]. In this study we hypothesize that auranofin, when administered as a single agent, will inhibit the Txnrd1 pathway *in vitro* and *in vivo* models of pancreatic cancer, and thereby prevent PDAC tumor growth and progression.

## MATERIALS AND METHODS

### Reagents

MTT, auranofin, dimethylsulfoxide (DMSO), and Polyethylene glycol (PEG) were obtained from Sigma-Aldrich, St. Louis, MO. Gemcitabine was donated from the pharmacy of The University of Texas, MD Anderson Cancer Center (UTMDACC). Ethyl Alcohol and injectable-H_2_O were obtained from Pharmaco-AAPER (Brookfield, CT) and Hospira (Lake Forest, IL), respectively. Plasmid pCDH-RFP-Luciferase was obtained from Dr. Mien-Chie Hung, from The Department of Molecular and Cellular Oncology, UTMDACC. Radioimmunoprecipitation protein assay (RIPA), PrestoBlue, and human Vascular Endothelial Growth Factor (VEGF) ELISA kit were purchased from Life Technologies (Grand Island, NY). Bio-Rad protein assay was obtained from Bio-Rad Laboratories Inc. (Philadelphia, PA). Polyvinylidene difluoride (PVDF) membranes and Cremophor EL were bought from Millipore (Billerica, MA). Antibodies to Txnrd1, HIF1α, and PARP were purchased from Abcam (Cambridge, MA). Antibodies to β-actin were purchased from Santa Cruz Biotechnology (Dallas, TX). Matrigel was obtained from Thermo Scientific, South Logan, UT, and Life Science Technologies, Tewksbury, MA, respectively. Gold analysis standard solutions of Au(III) (1000 mg L-1), hydrochloric acid (HCl) suprapure (36%), and nitric acid suprapure (65%), were purchased from Merck, Germany.

### Generation of Cell Lines and Xenografts

MD Anderson (MDA) patient-derived cell lines (PATC; Suppl. Table 1) and patient-derived tissue xenografts (PATX; Suppl. Table 2) were established from patients diagnosed with PDAC who underwent either pancreatectomy or metastasectomy (resected from liver, lung, or bone) over the period of 2010–2014, who consented for both surgery and tissue collection under Institutional Review Board (IRB) protocols: LAB07–0854, LAB00–396, and PA15–0176. Animal protocols were approved by the MD Anderson following The Animal Care and Use Form (ACUF) protocol 00001089-RN00. Eleven cell lines were derived from MDA xenografts which were named as follows: MDA-PATC43, 50, 53, 69, 76, 102, 108, 113, 121, 124, and 135, the first 3 were characterized on a previous report [22]. Seven out of the remaining 8 newly established cell lines were confirmed to be unique by DNA fingerprinting performed at the Characterized Cell Line Core Facility (UTMDACC), 5 of which were matched with patients (data not shown). Established commercial cell lines 293 T, BxPc-3, Panc-1, MiaPaCa-2, HPAF II, Hs-776-T, SW1990, AsPc-1, and PanO2, were obtained from ATCC (Manassas, VA). Cell lines and tissues were used from patients of both sexes.

Procedures for PDX establishment were followed as described on a previous publication [23]. Briefly, different generations of PDX were grown in bilateral subcutaneous areas of 6–8-week-old female NOD/SCID (F1 – first generation) or nude mice (F2-F4) obtained from Jackson Laboratory (Bar Harbor, ME). Tumors reaching 1–1.5 cm diameter were harvested following euthanasia under aseptic technique. Patient-derived tissue xenografts (PATX) were labeled according to the generation, F1-F4, and were used for drug sensitivity testing and histological analysis.

### Cell Viability Assay

The MTT cell proliferation assay was used as per the manufacturer's protocol to determine cell line viability. Each experiment consisted of 2–3 × 10^3^ cells/well seeded on a 96-well plate, followed by treatment 24 hours later with a series of six 1:10 dilutions of auranofin in 0.1% DMSO, with 0.1% DMSO as control. Hypoxia experiments (O_2_ < 1%) were done using a hypoxia chamber incubator (BioMedical Solutions Inc., Stafford, TX), and a nitrogen regulator (NuAire, Inc., Plymouth, MN). Absorbance was read at 570 nm, 72 hours after treatment, using the FLUOstar Omega plate reader (BMG Labtech, Offenburg, Germany) at the Core Facility, UTMDACC. Experiments were done in triplicates.

### Luciferase Transfection

The plasmid pCDH-RFP-Luciferase was transfected in MiaPaCa-2 and MDA-PATC53 cells as previously described by our group [24]. Briefly, pCDH-RFP-Luciferase plasmid recombinant viruses were generated by transient transfection of the packaging plasmids pMLg/pRRE, pRSV.rev, and pHCMV-G into 293 T cells. Virus-containing supernatant was collected after 72 hours to infect MiaPaCa-2. Infected cells were purified by RFP fluorescence-activated cell sorting at the Flow Cytometry and Cellular Imaging Facility, UTMDACC.

### Western Blot Assay

Western blot assay was performed following the previously reported protocol [25]. In brief, whole cell lysates were extracted using RIPA with 1X-protease inhibitor cocktail from: MDA-PATC53, MiaPaCa-2, SW199O, and AsPc-1, 24 hours following incubation with 0.1%D MSO and variable auranofin-doses ranging from 0 to 10 μmol/L. Protein concentration was determined using the Bio-Rad protein assay followed by loading 20 μg in 8–15% SDS polyacrylamide gels run at 96-Volts at room temperature. Gels were transferred overnight to PVDF membranes. Primary antibodies were incubated at 1:1,000 (PARP and HIF1α), and 1:500 (Txnrd1) at 4 °C overnight under rocking motion. Loading control (β-actin) was incubated at 1:5,000 for 2 hours at room temperature under rocking motion. Secondary antibodies were incubated at 1:5,000 for 2 hours at room temperature, under rocking motion. Protein bands were visualized using Kodak developer (Core Laboratories, UTMDACC) 1 minute after enhancer chemoluminescence reagent was added. Exposure times varied from 5 seconds to 5 minutes.

### Live Tissue Slice Assay

Live tissue slice assay (LTSA) was done following previously reported protocol [26]. In brief, 2–4 punch biopsies of 3–4 mm diameter were performed on an explanted PDX in a sterile 10 cm petri dish, then immediately placed in University of Wisconsin solution with 2% Penicillin/Streptomycin on ice (Bridge to Life, Columbia, SC). Cores were then transferred to 1% low melting point agarose gel and were cut into high-precision tissues slices of 200 μm thickness with a live tissue microtome filled with cold sterile PBS containing 2% penicillin/streptomycin (Krumdieck, Alabama Research and Development, Munford, AL). Tissue slices were transferred from the PBS to a 96-well plate with DMEM-10%FBS for *ex vivo* tissue culture and maintained at 37 °C and 5% CO_2_ on a plate shaker at 150 rpm. Slices were treated within 24 hours with auranofin 10 μmol/L and control. Tissue viability was determined 48–72 hours post-treatment using the PrestoBlue viability assay per the manufacturer's protocol. Absorbance was read at 570 nm using FLUOstar Omega reader (Core Facility, UTMDACC). Experiments were done in triplicates.

### Human Vascular Endothelial Growth Factor (VEGF) ELISA of PDX Tissue Slices

Fresh tissue slices of PDX79-F4 were cultured under normoxia and hypoxia for 6 hours and 24 hours post-treatment with auranofin 10 μmol/L and control (0.1% DMSO). Conditions were replicated in 4 wells, each containing a tissue slice 4 mm in diameter and 200 μm in thickness. Tissue culture media were collected and diluted in a 1:3 ratio for each condition. Diluted samples were used for the human VEGF ELISA following manufacturer's instructions. Results were normalized by tissue slide protein concentration and analyzed using ANOVA from GraphPad Prism.

### Generation of MiaPaCa-2-Luc + Orthotopic Mouse Model

Twenty 6-week old female nude mice were obtained from Jackson Laboratories. MiaPaCa-2-Luc + cells were grown in culture to an 80–90% confluence with confirmed luminescence *in vitro* using Xenogen-IVIS200 from Small Animal Imaging Facility (SAIF), UTMDACC. Cells were prepared in a 1:1 mixture of PBS: Matrigel to a concentration of 5 × 10^6^ cells/mL. Using aseptic technique for rodent survival as per IACUC standards, an incision was made in the left abdomen, the tail of the pancreas was exteriorized, and 2.5 × 10^5^ cells/50 μL of MiaPaCa-2-Luc + were slowly injected to the tail of the pancreas following a previously published protocol [27]. The peritoneum was closed with two 5–0 Vicryl interrupted stitches and skin was closed with metal clips. All mice survived surgery and formed tumors, monitored twice-a-week by bioluminescence imaging (BLI) acquisition with IVIS200 (Xenogen, Alameda, CA). Mice were randomly distributed among 4 groups (of *n* = 5): Vehicle, auranofin 5 mg/kg, 10 mg/kg, and 15 mg/kg. Neither pre-treatment average BLI (radiance) nor mouse weight (grams (g)) were significantly different among groups (data not shown). Vehicle solution was composed of 5% DMSO, 10% Cremophor EL, 12.5% PEG, and 15% Ethyl Alcohol, and 57.5% H_2_O. Auranofin solutions were calculated based on a 25 g mouse (pre-treatment average mouse weight was 23 g). Mice were weighed weekly. Treatment was started on post-injection day 21 on a Monday through Friday daily schedule, with intraperitoneal injections of 100–300 μL, corresponding to 5–15 mg/kg. Mice were anesthetized prior to treatment due to solution viscosity and to avoid organ injury or erroneous delivery route. Time of euthanasia was reached when abdominal distension and/or large tumor burden were present as per DVMS. Each mouse underwent a post-euthanasia necropsy for documentation of presence of ascites and/or gross abdominal cavity metastases, as well as for tissue collection. The experiment was ended once all mice reached criteria for euthanasia. Kaplan–Meier curves were constructed from the survival data.

### Gold Content Analysis

Measurements of gold content were performed in tissues from: MiaPaCa-2 (tumor, liver, and kidney), and MDA-PATC53 (tumors only), carried out with a Varian SpectrAA 880Z atomic absorption spectrometer equipped with a GTA 100 graphite furnace with Zeeman background correction system (GFASS). The gold hollow cathode lamp (Narva, Germany) was operated at 5 mA current. The absorbances were measured using the pyrolytically coated graphite tube at 242.8 nm with 0.2 nm spectral band pass. The temperature program applied in those determinations was: drying 110 °C (for 20 s), pyrolysis 900 °C (for 10s), and atomization 2,100 °C (for 5 s). Measurements were carried out by calibration with liquid standards. Samples of tissues were accurately weighed (mass range: 42.8–285 mg), and directly digested in glass auto sampler cups with 1.5 mL of aqua regia by gentle heating on a plate to almost dryness. The digestion process was repeated 3 times. The final residue was heated up to 800 °C with 1.0 mL of 1.0 M HCl. The gravimetric method was used to control the final volume due to relatively low tissue masses to be analyzed. Chemical blanks and tissue samples with addition of known amount of gold standard were analyzed as controls of the digestion procedure and measurement by GFAAS technique. The tissues samples chosen were spiked prior to the digestion; known concentrations of the gold standard were also used for the calibration. This approach was necessary because suitable biological reference material for gold certified is not commercially available. The gold recovery for such prepared samples was > 95%.

### Statistical Analysis

Experiments were performed in triplicates and values are presented as mean ± SEM. GraphPad Prism 6 was used for statistical analysis. T-tests were done to compare the difference between 2 groups and ANOVA to compare more than 2 groups. Survival statistics were presented as Kaplan–Meier curves.

#### RESULTS

##### Single Agent Auranofin Effectively Decreases Pancreatic Cancer Cell and Xenograft Tissue Slice Viability by Multiple Mechanisms

**Fig. 1:**
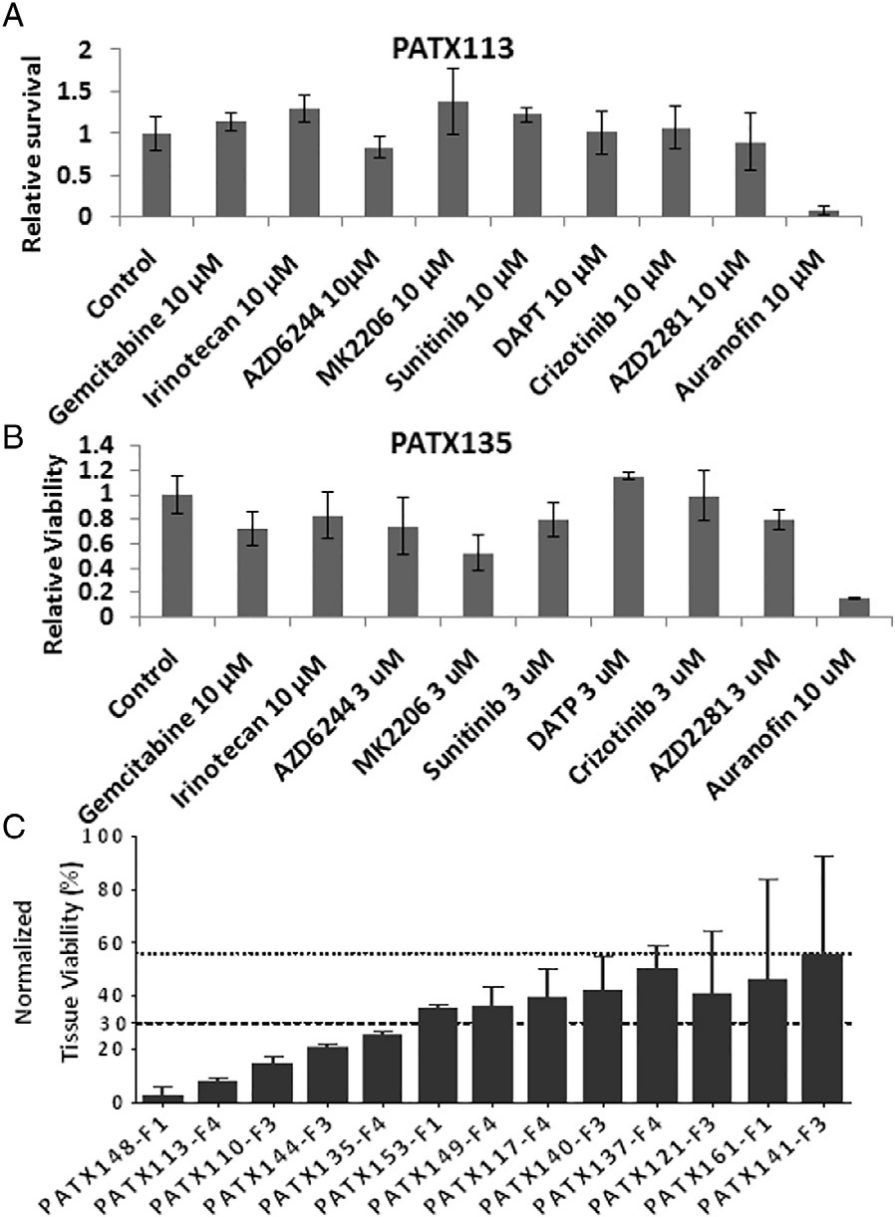
**Live tissue slice assay for chemosensitivities of pancreatic adenocarcinoma patient-derived xenografts.** (A and B) Representative figures showing the testing of a panel of FDA-approved or clinical testing agents on patient-derived xenograft tissue slices of PDAC using a live tissue slice assay (LTSA). Auranofin was noticed to be more active by decreasing tissue viability at a higher degree, when compared to other agents [Gemcitabine, Irinotecan, AZD6244 (MEK inhibitor), MK2206 (AKT inhibitor), Sunitinib (receptor protein-tyrosine kinase inhibitor), Dual Antiplatelet Therapy (DAPT), Crizotinib (ALK/c-Met/HGFR inhibitor), AZD2281 (PARP inhibitor) and untreated tumor xenograft tissue slices. (C) Normalized tissue viability was calculated for 13 tested tissue samples, and growth inhibition (%Inhibition = 100 – %Normalized Viability) induced by auranofin was found to be 44–95%.

**Fig. 2:**
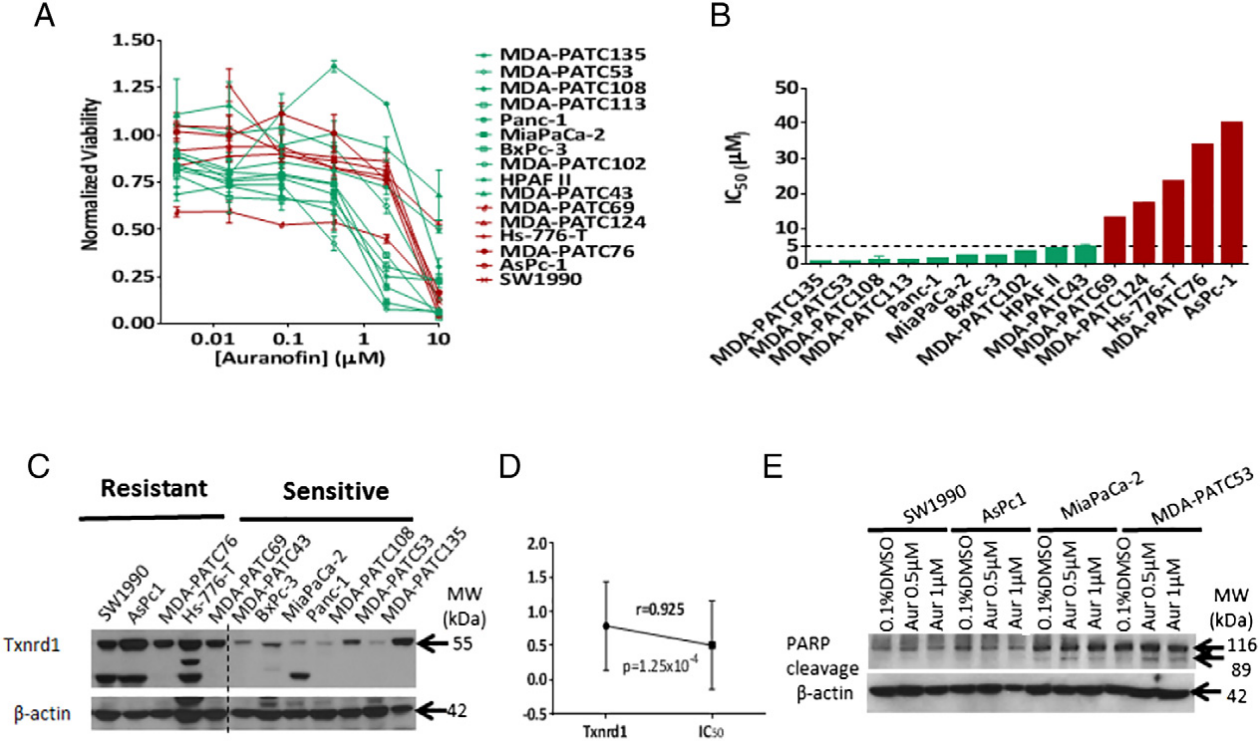
**Mechanisms of action of auranofin in human pancreatic adenocarcinoma models**. (A) MTT assay was performed to assess for cell viability 72 hours after auranofin treatment at different concentrations. (B) IC_50_ determination for 15 human PDAC cell lines, 10 of which were defined as auranofin-sensitive (in green) based on IC_50_ < 5 μmol/L. The remaining cell lines were defined as auranofin-resistant (in red) due to IC_50_ ≥ 5μmol/L. (C) Western blot for Txnrd1 and baseline expression was performed for a panel of 13 human PDAC cell lines. Dotted vertical line divides cell lines in 2 subgroups based on IC_50_. (D) Txnrd1 protein expression and IC_50_ from human PDAC cell lines were found with a statistically significant positive correlation. (E) Western blot analysis was performed following low-dose (0.5–1 μmol/L) auranofin treatment showing higher PARP cleavage for MiaPaCa-2 and MDA-PATC53 (auranofin-sensitive), unlike auranofin-resistant cell lines SW1990 and AsPc-1. β-Actin was used as a loading control, and 0.1% DMSO used as the control treatment.

**Fig. 3:**
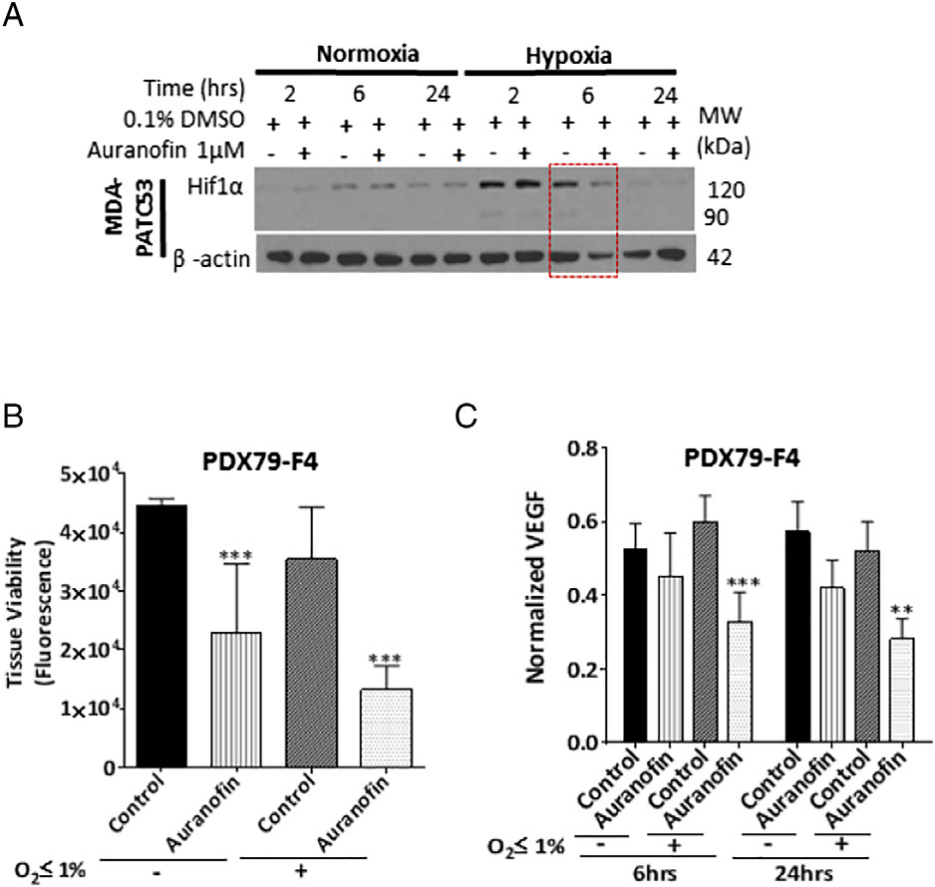
**Hypoxia enhances antiangiogenesis mechanism of auranofin in human PDAC**. Inhibition of Hif1α was observed at 6 hours post-treatment for MDA-PATC53 under hypoxia (O_2_ < 1%), which was not present during normoxia. (B) Treatment of PDX79-F4 with auranofin 10 μmol/L induced a statistically significant inhibition of tissue viability under both normoxia and hypoxia, and trended towards a bigger difference under hypoxia. (C) Human VEGF ELISA of tissue culture media from tissue slices of PDX79 revealed inhibition of VEGF secretion in all auranofin-treated groups, but became statistically significant under hypoxia at 6 and 24 hours.

In a live tissue slice assay (LTSA), auranofin showed the highest suppression of relative viability of *ex vivo* PDAC tumor slices out of a panel of 9 approved (e.g., Gemcitabine, Irinotecan), and on-trial anticancer agents (e.g., AZD6244-MEK inhibitor, MK2206-AKT inhibitor, Sunitinib-receptor protein-tyrosine kinase inhibitor, Dual Antiplatelet Therapy (DAPT), Crizotinib-ALK/c-Met/HGFR inhibitor, AZD2281-PARP inhibitor) on patient derived xenografts MDA-PATX-113 and MDA-PATX-135 (Fig. 1A-B). Normalized tissue viability was calculated for thirteen tested tissue samples, and growth inhibition (%Inhibition = 100 – %Normalized Viability) induced by auranofin was found to be 44–95% (Fig. 1C). The majority (10/15; 67%) of the human PDAC cell lines tested were found to be auranofin-sensitive based on IC_50_ ≤ 5 μmol/L (Fig. 2A-B; Suppl. Table 3). These cell lines were derived from both primary and metastatic (e.g., liver, lung, ascites (AsPc1, and HPAF II), and bone; Suppl. Table 1) sites. Txnrd1 expression was overexpressed in resistant cell lines (Fig. 2C), resulting in a statistically significant positive correlation between Txnrd1 and IC_50_ (Fig. 2D). Treatment with low-dose auranofin (0.5–1 μmol/L) was found to induce apoptosis by showing higher PARP-cleavage (Fig. 2E) among sensitive cell lines when compared to resistant. MDA-PATC53 exhibited an inhibition of HIF1α expression at 6 hours (Fig. 3A) under hypoxia when compared to normoxia. Treatment of PDX79-F4 with auranofin 10 μmol/L induced a statistically significant inhibition of tissue viability (Fig. 3B) under both normoxia and hypoxia, and trending towards a bigger difference under hypoxia. VEGF secretion from xenograft tissue slices from PDX79-F4 were significantly suppressed at 6 and 24 hours after auranofin 10 μmol/L treatment under hypoxia (Fig. 3C), when compared with normoxia controls.

##### Auranofin Presents a Survival Advantage in Nude Mice with MiaPaCa-2 Pancreatic Tumors and Inhibits Metastasis in a Dose-Dependent Fashion

**Fig. 4:**
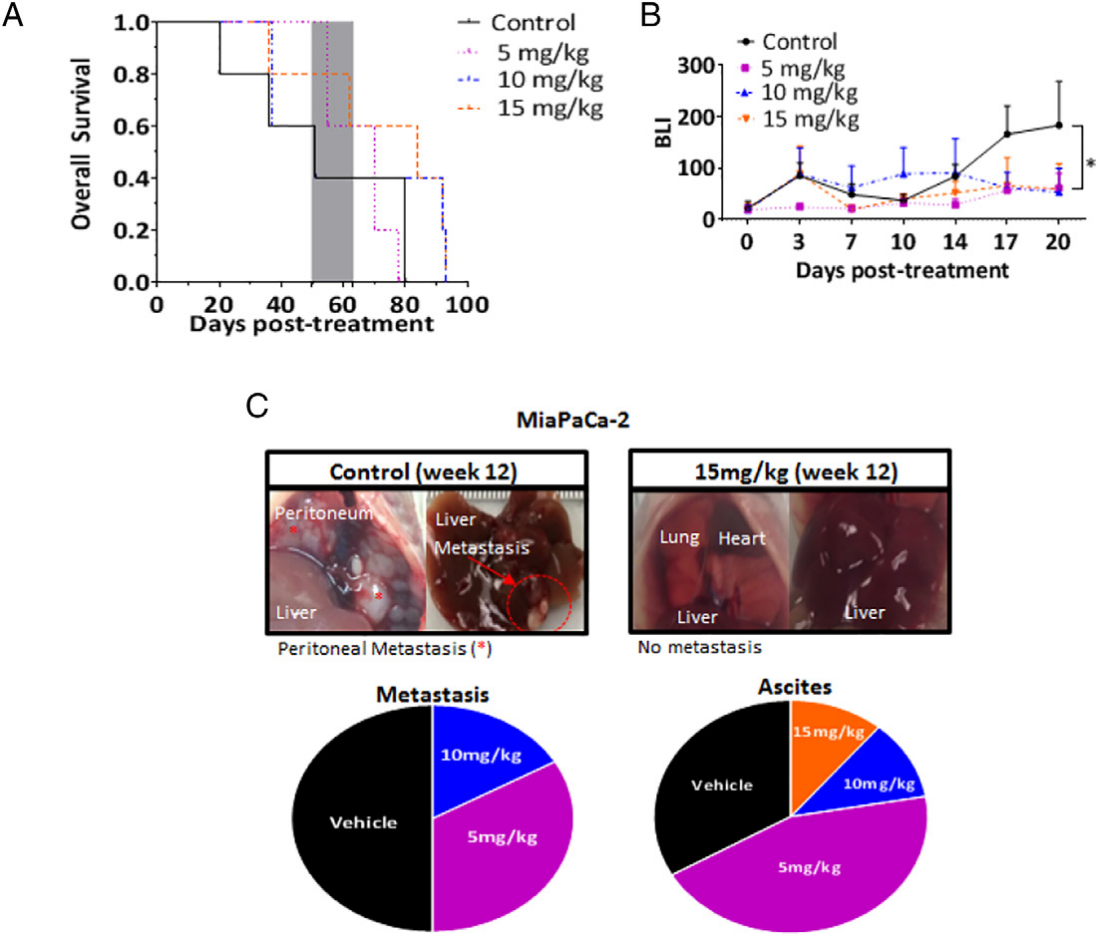
**Auranofin delays progression and prolongs survival of nude mice with MiaPaCa-2 tumors**. MiaPaCa-2-Luc + cells (2.5 × 10^5^ cells in 1:1 PBS to Matrigel ratio) were orthotopically implanted in twenty 6-week-old female nude mice with 100% take rate confirmed by BLI (not shown) 2-weeks post-implantation. Mice were randomized (5 mice/group), and treated Monday to Friday once a day with: Vehicle, Auranofin 5, 10, or 15 mg/kg as intraperitoneal injections. Mice were euthanized when: abdominal distension or large tumor burden (> 2 cm) occurred. Mice treated with 15 mg/kg auranofin trended towards longer survival (*P* = .095). (B) BLI (in radiance × 10^6^ p/sec/cm^2^/sr) was recorded twice a week over the first 20 days, until the first mortality occurred. Groups treated with 10 and 15 mg/kg at day 20 were found to have significantly less luminescence compared to the control group, implying lower tumor burdens (*). (C) Representative photos showing the lack of gross solid organ metastasis for mice in the 15 mg/kg group versus the control treatment group. Total metastatic event occurrence (lower left) and ascites (lower right) were recorded during necropsy, with proportions showing less of either as treatment dosage increased.

**Fig. 5:**
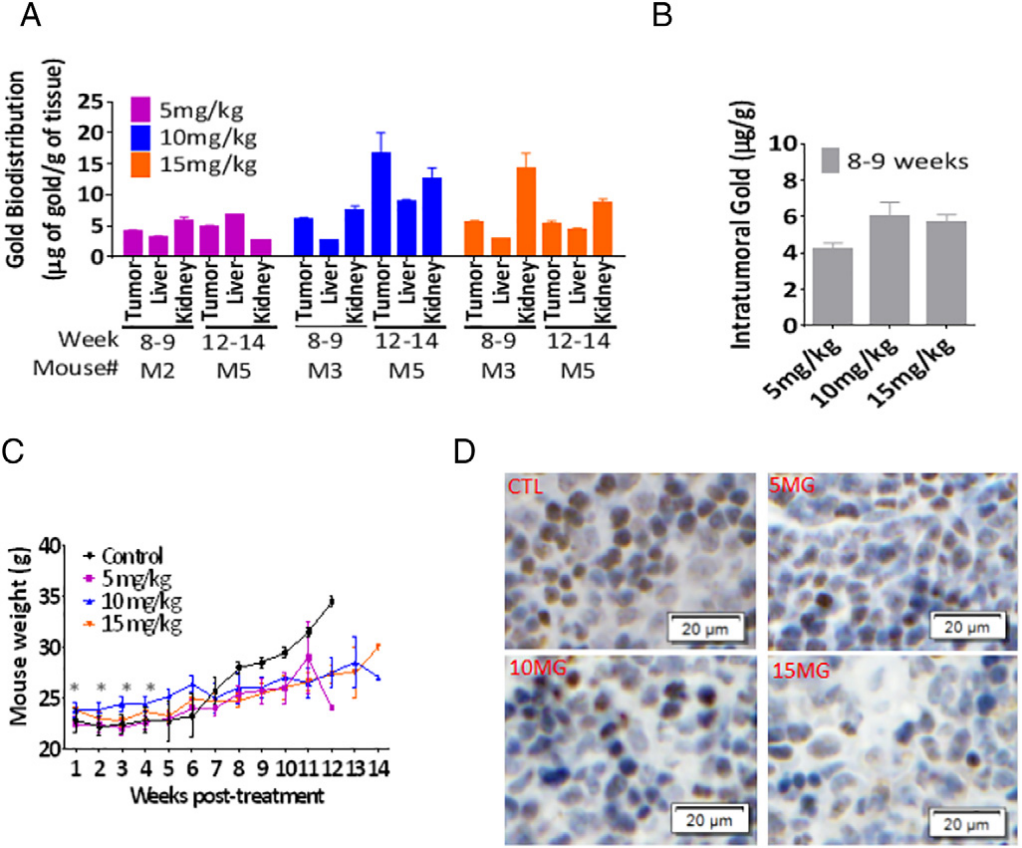
**Auranofin biodistribution, mouse weight change, and MiaPaCa-2 tumor Hif1α expression**. Gold content was quantified from different tissues (tumor, liver, and left kidney) retrieved at the time of death (8–9 weeks and 12–14 weeks) for one mouse (M) per group as shown in the figure, to demonstrate gold biodistribution. Vehicle group was omitted due to levels below level of detection. (B) Intratumoral gold (μg of gold per g of tissue) was analyzed from tissue harvested at the time of death. (C) Mice weights were recorded weekly and were statistically significant different than control over the first 4 weeks post-treatment versus control (*). (D) Representative figure of Hif1α tissue expression for MiaPaCa-2 tumors under different doses of auranofin treatment, indicating less expression of Hif1α with higher doses of auranofin.

There was a 100% tumor take rate using this model. A survival study using orthotopically implanted MiaPaCa-2-Luc + cells showed 15 mg/kg auranofin to be the optimal dose due to a trend towards survival benefit post-treatment initiation versus control (Median Survival of 12 versus 8 weeks, respectively; *P* = .095; Fig. 4A, Suppl. Table 4). The 15 mg/kg auranofin group had not only a statistically significant lower tumor burden (as measured with bioluminescence; Fig. 4B) 20 days post-treatment when compared to control, but exhibited a complete suppression of gross abdominal metastasis and a lower occurrence of ascites (Fig. 4C) when compared to other treatment groups. The overall frequency of liver metastasis found at the time of death was 30% (6/20). Of those 50% were in the control group, 33% in the 5 mg/kg, 17% in the 10 mg/kg, and none in the 15 mg/kg auranofin group. There was a dose dependent occurrence of gross abdominal distant organ metastasis (Fig. 4C). The overall occurrence of ascites was 45% (9/20). Of these 33% were found in the control group versus 44% in the 5 mg/kg, and 11% in both 10 and 15 mg/kg auranofin groups (Fig. 4C). The biodistribution of auranofin was determined by measurements of gold content in one mouse per group from: tumor, liver, and left kidney, at 2 different periods post-necropsy (8–9 weeks and 12–14 weeks; Fig.5A). Intratumoral gold (in μg of gold/g of tissue) was determined from tissue retrieved post-necropsy at the time-of-death (Fig. 5B) from the time period depicted in gray in Fig. 4A (weeks 8–9). There was a statistically significant weight gain exhibited for all treatment groups versus control during the first 4 weeks post-treatment (Fig. 5C). Immunohistochemistry of tumor tissue for HIF1α demonstrated an auranofin-induced decreased expression when compared to control (Fig. 5D).

#### **DISCUSSION**

Methods to inhibit progression of pancreatic adenocarcinoma are urgently needed. The most frequent site of metastases are the liver, peritoneum, lung, bones, and adrenal glands [28]. In this study, we demonstrated the anti-cancer activity of auranofin in *several* models of pancreatic adenocarcinoma. Importantly, the anti-cancer activity translated into decreased metastasis in a clinically relevant *in vivo* model. Additionally, this study supports apoptosis, inhibition of HIF1α and VEGF secretion as molecular consequences associated with antitumoral effect and identifies Txnrd1 expression as a potential biomarker of auranofin-resistance: a finding consistent with previously described reports [29].

In our models, auranofin prevented progression of human PDAC by showing antitumor effect at the primary site and complete suppression of distant organ metastasis at a dose of 15 mg/kg IP. Previous studies reported optimal doses of ≥ 2 μmol/L for Pa Tu II cell line and 12 mg/kg IP for an immunocompetent leukemia mouse model, which are consistent with our observations [21], [30]. While other investigators have demonstrated a delay in lung metastasis in an osteosarcoma orthotopic model, this is the first report testing auranofin against pancreatic cancer animal model [10]. Using techniques developed for the determination of tissue gold biodistribution at the time of death, we verified that organic gold compounds were present in variable amounts in different tissues (Fig. 5A), which could account for the anti-metastatic effects in our nude mice model (Fig. 4C; Suppl. Fig. 4).

Hypoxia is commonly found within pancreatic tumors, and larger regions of intra-tumoral hypoxia within PDAC tumors correlates with diminished survival [31]. While the exact mechanism is unknown, it is hypothesized that the tumor inhibitory effect of auranofin lies in its ability to inhibit the cancer cell response to hypoxia. Our experimental results demonstrated that auranofin decreased hypoxia-driven VEGF secretion (Fig. 3C) as well as HIF1α expression in tumor cells (Fig. 3 **and** Fig. 5D), and hypoxic tumor samples were more sensitive to the apoptotic effects of auranofin during *ex vivo* experiments (Fig. 3B). It is not clear why inhibition of HIF1α occurs at 6 hours and not at 2 hours (Fig. 3A) however we can speculate that this may be because the inhibitory effect of auranofin on HIF1α occurs at a specific concentration level of associated factors such as reactive oxygen species (ROS), which stabilize HIF1α and protect it from degradation. It has been shown that hypoxia-driven ROS production causes autocrine production of VEGF which in turn stabilizes HIF-1 through an Akt-dependent mechanism in melanoma cells [32]. Auranofin is an inhibitor of multiple points of the PI3K/akt/mTOR pathway [6], and this may factor in the ability of auranofin to stop this auto-feedback loop. Inhibition of HIF1α and VEGF has been demonstrated in experiments with thioredoxin redox inhibitors (1-methylpropyl 2-imidazolyl disulfide, and pleurotin) [19], and we found that resistant PDAC cells, as demonstrated by IC_50_, uniformly expressed Txnrd1 at high levels. The Trx system protects normal cells from injury and has been associated with cancer cell drug resistance [14], [33], [34], [35], [36], [37], [38], [39]. Inhibition of Txnrd1 to increase ROS is being explored as a radiosensitizer in chemo-radiation regimens [9]. Combining these agents with auranofin could represent a strategy to overcome the hypoxic tumor microenvironment in PDAC.

The mechanism for the presence of gold compounds in the tissue and inhibition of metastatic tumor formation at these sites is not explained in this report. Levels of auranofin in solid organs have not been measured in humans to our knowledge. An early study of auranofin showed that blood gold levels in patients reached past 1.1 μg/ml only by taking treatment of 3 mg twice a day for 3 weeks, then 3 times a day for 9 weeks, for a total of 12 weeks of treatment, which is a much lower dosage than our 5–15 mg/kg mouse regimen [40]. The measurement of intra-tumoral gold concentration in pancreatic cancer xenografts is also described for the first time in this study, and further studies can be conducted to determine optimal intra-tumoral gold concentrations to elicit the best response.

Additional weaknesses of this study include small animal group sizes and lack of testing an immunocompetent mouse model. Future directions include testing different dosages, different delivery routes such as intravenous or oral administration, and treatment timing regimens and investigating whether there would be treatment synergy with auranofin and other chemotherapeutics or radiation in PDAC and other solid tumors. Further testing of auranofin on an immunocompetent animal model will be of particular interest in order to elucidate its immunomodulatory roles with regard to tumor formation.

Over the last years there have been more efforts towards repurposing FDA-approved drugs as anticancer agents along with clinical trials that could lead to a significant survival benefit for this patient population [15], [41]. The study of gold-compounds anti-cancer properties could lead to the development of auranofin-like compounds as new treatments, and these data support further investigation of auranofin in this capacity. As this drug is already FDA-approved and has been used for decades with a well-studied safety profile, we think it is reasonable to advocate for a Phase 0 clinical trial of off-label use of auranofin for pancreatic cancer patients. Other preclinical studies have demonstrated the effectiveness of auranofin in preventing metastases, so this drug may be useful as an adjuvant treatment after primary tumor resection to prevent distant recurrences. Also the fact that it is bioavailable through oral administration will make it an attractive chemotherapeutic option for patients, similar to the advantage.

## Author Contribution

Mayrim V. Rios Perez, MD^a^, conceived and designed the analysis, collected the data, contributed data or analysis tools, performed the analysis, wrote the paper. David Roife, MD^b^, conceived and designed the analysis, performed the analysis, wrote the paper. Bingbing Dai, PhD^c^, conceived and designed the analysis, collected the data, contributed data or analysis tools, performed the analysis. Michael Pratt, BA^d^, collected the data, contributed data or analysis tools, performed the analysis. Ryszard Dobrowolski, PhD^e^, conceived and designed the analysis, collected the data, contributed data or analysis tools, performed the analysis. Ya'an Kang, MD PhD^c^, conceived and designed the analysis, collected the data, contributed data or analysis tools. Xinqun Li, MD PhD^c^, conceived and designed the analysis, collected the data, contributed data or analysis tools. Jithesh J. Augustine, MS^c^, conceived and designed the analysis, collected the data, contributed data or analysis tools. Rafal Zielinski, PhD^f^, conceived and designed the analysis, collected the data, contributed data or analysis tools. Waldemar Priebe, PhD^f^, conceived and designed the analysis, collected the data, contributed data or analysis tools. Jason B. Fleming, MD^b^ conceived and designed the analysis, performed the analysis, wrote the paper.

#### CONFLICT OF INTEREST

The authors disclose no potential conflicts of interest.

#### FUNDING SOURCES

NIH T32CA009599 (M.V.R.P and D.R.), and P30CA016672 (Core Laboratory, MD Anderson Cancer Center).

## Appendix A Supplementary data

Supplementary material Image 1
