# Supplementary material for: Antineoplastic effects of auranofin in human pancreatic adenocarcinoma preclinical models
Source: Surg Open Sci. 2019 Jul 3;1(2):56–63. doi: 10.1016/j.sopen.2019.05.004 (PMC8083010; doi:10.1016/j.sopen.2019.05.004)
Supplement: Supplementary file 1 — Supplementary material [file mmc1.docx]

**Supplementary Table 1. MDA cell lines used for *in vitro* drug testing**

| Cell Line ID | Passage No. | F0 Tumor Origin |
| --- | --- | --- |
| MDA-PATC43 | P7 | Pancreas |
| MDA-PATC50 | P7 | Pancreas |
| MDA-PATC53 | >P10 | Metastasis: Liver |
| MDA-PATC69 | >P10 | Pancreas |
| MDA-PATC76* | P5 | Metastasis: Lung |
| MDA-PATC102 | P8 | Pancreas |
| MDA-PATC108 | P5 | Pancreas |
| MDA-PATC113* | P3 | Metastasis: Bone |
| MDA-PATC121 | P2 | Metastasis: Liver |
| MDA-PATC124 | P2 | Pancreas |
| MDA-PATC135* | P3 | Metastasis: Bone |

F0 Tumor Origin refers to patient’s tissue of origin prior to implantation in mice; P stands for cell passage number (No.) in culture.*Cases from the same patient but different sites (PATC113 and 135 are from vertebra and femur, respectively).

**Supplementary Table 2. MDA-Tissue xenografts used for drug testing**

| Tissue ID | Generation No. | F0 Tumor origin |
| --- | --- | --- |
| PATX148 | F1 | Metastasis: Liver |
| PATX113* | F4 | Metastasis: Bone |
| PATX110 | F3 | Pancreas |
| PATX144 | F3 | Pancreas |
| PATX135* | F4 | Metastasis: Bone |
| PATX153 | F4 | Metastasis: Liver |
| PATX149 | F4 | Metastasis: Liver |
| PATX117 | F4 | Pancreas |
| PATX140 | F3 | Pancreas |
| PATX137 | F4 | Pancreas |
| PATX121 | F3 | Metastasis: Liver |
| PATX161 | F1 | Pancreas |
| PATX141 | F3 | Pancreas |
| PATX79 | F4 | Pancreas |

F refers to the generation or tumor passage number (No.) in mice. *Cases from the same patient but different sites (PATX113 and 135 are from vertebra and femur, respectively).

**Supplementary Table 3. IC_50_ for 15 human PDAC cell lines**

| Cell line | IC_50_ (µM) |
| --- | --- |
| MDA-PATC135 | 0.728 |
| MDA-PATC53 | 0.760 |
| MDA-PATC108 | 1.04 |
| MDA-PATC113 | 1.09 |
| Panc-1 | 1.50 |
| MiaPaCa-2 | 2.34 |
| BxPc-3 | 2.39 |
| MDA-PATC102 | 3.47 |
| HPAF II | 4.46 |
| MDA-PATC43 | 4.77 |
| MDA-PATC69 | 13.3 |
| MDA-PATC124 | 17.3 |
| Hs-776-T | 23.6 |
| MDA-PATC76 | 33.8 |
| AsPc-1 | 40.2 |

**Resistant-------------------------------------------------------> Sensitive**

**Supplementary Table 4. Auranofin prolongs survival of nude mice with MiaPaCa-2 tumors**

|  | MS (weeks) | P value | OS (weeks) |
| --- | --- | --- | --- |
| Control | 8 | N/A | 12 |
| 5mg/kg | 10 | 0.781 | 12 |
| 10mg/kg | 8 | 0.307 | 14 |
| 15mg/kg | 12 | 0.095 | 14 |
